# Impact of dietary *Chlorella vulgaris* and feed enzymes on health status, immune response and liver metabolites in weaned piglets

**DOI:** 10.1038/s41598-022-21238-9

**Published:** 2022-10-07

**Authors:** Cátia F. Martins, Paula A. Lopes, Mariana Palma, Rui M. A. Pinto, Mónica Costa, Cristina M. Alfaia, José M. Pestana, Diogo Coelho, David M. Ribeiro, Ivan Viegas, André M. Almeida, João P. B. Freire, José A. M. Prates

**Affiliations:** 1 CIISA - Centre for Interdisciplinary Research in Animal Health, Faculty of Veterinary Medicine, University of Lisbon, 1300-447 Lisbon, Portugal; 2 LEAF - Linking Landscape, Environment, Agriculture and Food, Higher Institute of Agronomy, University of Lisbon, 1349-017 Lisbon, Portugal; 3 Department of Life Sciences, Centre for Functional Ecology, University of Coimbra, 3000-456 Coimbra, Portugal; 4 iMed.UL, Faculty of Pharmacy, University of Lisbon, Avenida Professor Gama Pinto, 1649-003 Lisbon, Portugal; 5 JCS - Laboratório de Análises Clínicas Dr. Joaquim Chaves, Avenida General Norton de Matos, Miraflores, 1495-148 Algés, Portugal

**Keywords:** Animal physiology, Metabolomics

## Abstract

In this study, we analysed the impact of dietary inclusion of *Chlorella vulgaris* and carbohydrases on general health, redox status, immune response, liver lipids and metabolites in weaned piglets. Forty-four male piglets were allocated into four diets: control (*n* = 11), CH (control diet with 5% CH, *n* = 10), CH+R (control diet with 5% CH plus 0.005% Rovabio Excel AP, *n* = 10), and CH+M (control diet with 5% CH plus 0.01% of a pre-selected four-CAZyme mixture, *n* = 11). After 15 days of trial, animals were slaughtered and samples of blood and liver collected. Spectrophotometry methods and commercial kits were used to determine blood parameters and gas and liquid chromatography for hepatic fatty acid and chlorophylls profiles, respectively. While total, LDL- and VLDL-cholesterol were increased by CH, the opposite was recorded for HDL-cholesterol (*p* < 0.001). Piglets fed CH-based diets presented an increase of IgG and a decrease of IgM (*p* < 0.001) which along with lymphocytes exacerbation contributed for piglets’ survival after weaning. *n*−6 PUFA were reduced in piglets fed CH and the opposite occurred for *n*−3 PUFA (*p* < 0.001), thus benefiting *n*−6/*n*−3 ratio in the liver. Chlorophylls amount was not changed by the use of Rovabio or enzymatic mixture. The discriminant analysis applied to hepatic parameters revealed a clear separation between control and CH-based diets but failed to discriminate feed enzymes. Our findings indicate health promoting effects of CH as feed ingredient in piglets’ nutrition at weaning, without negatively impacting on animals’ performance.

## Introduction

The post-weaning phase is one of the most critical periods in swine production^1^. Indeed, animals have to face several adverse factors: complex social changes related to the separation from their mothers and littermates, changes in feeding and environment, and an immature immune system^2,3^. Therefore, at the weaning phase, piglets are particularly susceptible to digestive and respiratory pathologies resulting from the imbalance between animals’ immunity and environmental stress^2,3^. The use of antibiotics for preventive or therapeutic purposes of these pathologies is strongly discouraged, thus it is crucial to apply different strategies to reduce or prevent their use. A nutritional strategy that has received increased attention is the use of prebiotics. Specifically, Lui et al.^4^ focused on the influence of prebiotics on gut health in pigs, highlighting the positive modification in intestinal microbiota and the decrease in enteric diseases in pigs. These authors also suggested that prebiotics impact the immune system but argued that more research is needed to prove these effects^4^. Microalgae are known for their prebiotic properties as recently reviewed^5^. Indeed, microalgae prebiotics effects should not be restricted to their polysaccharides and lignin, but should be extended to their monosaccharides, enzymes, polyunsaturated fatty acids (PUFA), peptides, polyphenols and alcohols^6^.

The use of whole microalgae in animal diets has additionally been studied as an alternative in monogastric feeding, mostly as a supplement^7–10^ but, in recent years, also as an ingredient^11–14^ approved by the European Union regulation. *Chlorella vulgaris* (CH) is one of the widely used microalgae, expanding its biomass use for animal feeding, among other purposes. It is characterized by relevant contents of crude protein, crude fat and carbohydrates, with respectively, 50–60%, 13–21% and 18–28% of dry matter^15^. In fact, the enriched concentrations of *n*−3 long-chain polyunsaturated fatty acids (LC-PUFA), vitamins, minerals, carotenoids, other pigments and bioactive compounds by CH represent a potential resource with well-known beneficial health implications for both animals and humans^16^. However, microalgae have recalcitrant cell walls, making them indigestible by the monogastrics. In accordance, the development of new technologies to improve microalgae nutrient utilization is absolutely needed in order to foster the cost-effective use of microalgae for the feed industry^17,18^. Carbohydrates-active enzymes (CAZymes) have been investigated, in several in vitro nutritional studies, as being able to degrade the recalcitrant cell wall of microalgae, improving their nutritional value for monogastrics feeding and allowing their use at higher incorporation levels in pig and poultry diets. Recently, Coelho and colleagues^19^ demonstrated the potential of a novel four-CAZyme mixture to disrupt the recalcitrant cell wall of CH. It is possible that the combination of microalgae and enzymes, in addition to improve nutrient digestibility, could also contribute to increase the prebiotic effect, as suggested by several authors^4,6^. This might be an interesting alternative to the use of antibiotics at the weaning phase of piglets due to the formation of protective prebiotics in the intestine, which is in line with EU recommendations and policies on antibiotic resistance and use in animal production^6^.

Currently, the knowledge about the effects of microalgae on the general health status and hepatic metabolism of piglets is practically non-existent. Nuclear Magnetic Resonance (NMR) techniques have proven to be important tools to a comprehensive overview on animal physiology and production^20^ and may be useful in the identification of metabolites associated with hepatic metabolism. As mentioned in Madeira et al.^21^, there is a pressing need to have such information^22^. Thus, we hypothesized that microalgae, mainly in combination with CAZymes, would contribute to improve the health status and metabolic condition of piglets during the weaning period. Therefore, the aim of our study was to assess the effect of 5% of dietary CH, individually or combined with two feed enzymes (the commercially available Rovabio Excel AP and the four-CAZyme mixture pre-selected by Coelho et al.^19^) on blood biochemical markers, immune function (leucocytes and immunoglobulins), oxidative status (serum antioxidant markers and liver antioxidant diterpenes and carotenoids), and hepatic lipids and metabolomics in weaned piglets.

## Results

### Impact of *Chlorella vulgaris* in piglets’ zootechnical performance

**Figure 1:**
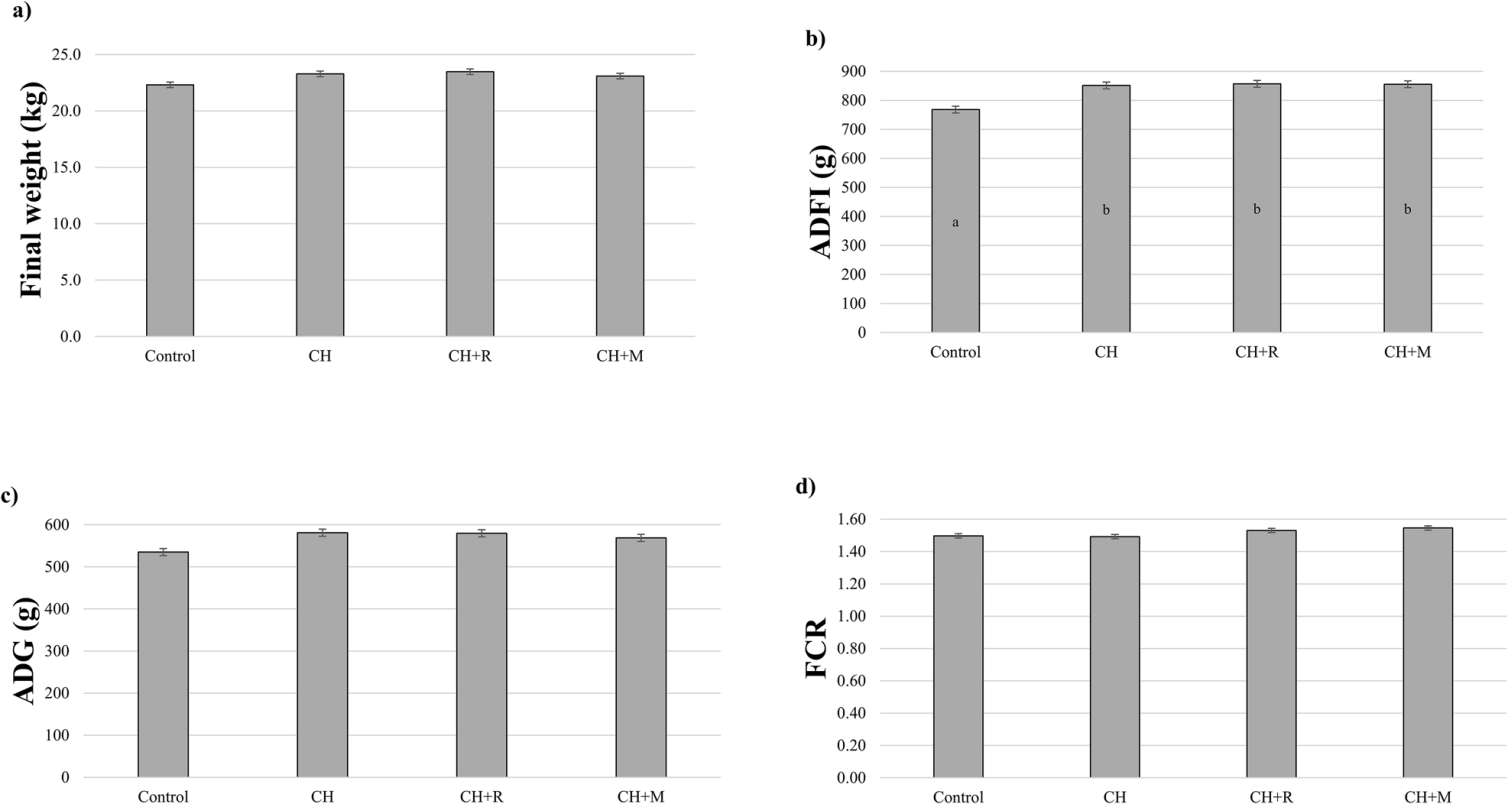
Influence of experimental diets on growth performance variables of piglets. (**a**) final body weight (kg), (**b**) ADFI—average daily feed intake (g), (**c**) ADG—average daily gain (g) and (**d**) feed conversion ratio. Dietary treatments: Control—control diet; CH—5% *Chlorella vulgaris* diet; CH+R—5% *Chlorella vulgaris* diet supplemented with 0.005% Rovabio Excel AP; CH+M—5% *Chlorella vulgaris* diet supplemented with 0.01% enzymatic mixture. ^a,b^Values with different superscripts differ significantly at *p* ≤ 0.05.

Figure 1 shows the influence of experimental diets on production performance of piglets. Final body weight (Fig. 1a), average daily gain (ADG) (Fig. 1c) and feed conversion ratio (FCR) (Fig. 1d) were unaltered by diets. In turn, ADFI (Fig. 1b) was increased by CH with or without feed enzymes (*p* < 0.05) during 15 days of experimental trial.

### Influence of experimental diets on blood parameters

Table 1 presents data on blood metabolites of piglets fed on CH with or without feed enzymes. Major variations were observed across haematology, serum biochemical markers, immunoglobulins and redox status. White blood cells count was higher in piglets fed the combination of CH and exogenous enzymes, when compared to the control animals (*p* = 0.004). Concerning the leucogram, lymphocytes (*p* < 0.001) and thrombocytes (*p* < 0.001) were increased in piglets fed the combination of CH and the enzymatic mixture relative to the other experimental groups. The opposite effect was observed for granulocytes (*p* < 0.001), which were decreased in piglets fed the combination of CH and the enzymatic mixture relative to the other experimental groups. Monocytes were unchanged by diets. Red blood cells (*p* = 0.001) and haemoglobin (*p* < 0.001) reached the highest values in piglets fed the combination of CH and feed enzymes in relation to the control group and CH alone. Total lipids (*p* < 0.001), triacylglycerols (TAG) (*p* < 0.001), total cholesterol (*p* < 0.001), LDL-cholesterol (*p* < 0.001), VLDL-cholesterol (*p* < 0.001), urea (*p* < 0.001) and creatinine (*p* < 0.001) were increased by CH combined with the enzymatic mixture relative to the other experimental groups. Conversely, the combination of CH and Rovabio increased HDL-cholesterol (*p* < 0.001) relative to the other experimental groups. Total protein did not change across diets. While glucose remained unchanged by diets, insulin reached the lowest value in piglets fed CH and the enzymatic mixture relative to the control (*p* = 0.006). The insulin resistance index (HOMA-IR) followed the same trend (*p* = 0.016). ALT was increased in piglets fed CH and the enzymatic mixture relative to the control and CH alone (*p* < 0.001) whereas AST (*p* < 0.001), ALP (*p* < 0.001) and GGT (*p* < 0.001) were increased in piglets fed Rovabio combined with CH compared to the other experimental groups.

**Table 1:** Influence of experimental diets on blood parameters of piglets.

	Diets				SEM	*p* value
	Control	CH	CH+R	CH+M		
**Haematology**						
White blood cells (× 10^9^/L)	15.2^a^	17.4^ab^	19.7^b^	20.7^b^	1.14	0.004
**Leucogram (% white blood cells)**						
Granulocytes	47.9^a^	42.9^a^	44.0^a^	36.4^b^	1.46	< 0.001
Lymphocytes	47.4^a^	53.5^b^	53.0^ab^	59.9^c^	1.61	< 0.001
Monocytes	4.71	3.63	3.00	3.75	0.706	0.364
Red blood cells (× 10^12^/L)	6.28^a^	6.43^a^	7.09^ab^	7.39^b^	0.217	0.001
Haemoglobin (g/L)	107^ab^	106^a^	116^bc^	120^c^	2.72	< 0.001
Thrombocytes (× 10^9^/L)	264^a^	305^a^	392^b^	472^c^	18.0	< 0.001
**Serum metabolites**						
Total lipids^1^ (g/L)	2.47^a^	2.66^b^	2.79^b^	3.63^c^	0.036	< 0.001
TAG^2^ (mg/L)	322^a^	399^ab^	447^b^	995^c^	22.4	< 0.001
Total cholesterol (mg/L)	505^a^	578^b^	637^b^	741^c^	16.3	< 0.001
HDL-cholesterol^3^ (mg/L)	148^a^	166^a^	267^c^	224^b^	10.0	< 0.001
LDL-cholesterol^4^ (mg/L)	336^b^	372^c^	304^a^	401^c^	7.82	< 0.001
VLDL-cholesterol^5^ (mg/L)	64.4^a^	79.8^ab^	89.4^b^	199^c^	4.49	< 0.001
Glucose (mg/L)	1193	1234	1201	1092	39.5	0.068
Insulin (mU/L)	1.45^a^	1.18^ab^	1.18^ab^	0.683^b^	0.162	0.006
HOMA-IR^6^ (mmol/L × μU/mL)	0.801^a^	0.665^ab^	0.616^ab^	0.339^b^	0.098	0.016
Urea (mg/L)	248^a^	255^a^	248^a^	428^b^	12.9	< 0.001
Creatinine (mg/L)	7.19^a^	7.57^a^	7.47^a^	10.2^b^	0.311	< 0.001
Total protein (g/L)	62.4	60.4	61.3	60.5	0.658	0.110
**Serum hepatic markers (U/L)**						
ALT^7^	23.5^a^	25.2^a^	27.4^ab^	30.7^b^	1.09	0.001
AST^8^	23.8^a^	23.0^a^	31.5^b^	19.7^a^	1.23	< 0.001
ALP^9^	185^a^	175^a^	257^b^	185^a^	6.20	< 0.001
GGT^10^	15.1^a^	16.5^a^	23.1^b^	16.4^a^	0.980	< 0.001
**Serum immunoglobulins**						
IgA^11^ (mg/L)	30.6	28.6	36.0	32.2	2.33	0.159
IgG^12^ (g/L)	0.507^a^	0.658^b^	0.750^bc^	0.776^c^	0.026	< 0.001
IgM^13^ (g/L)	0.766^a^	0.496^b^	0.696^a^	0.579^b^	0.024	< 0.001
**Serum antioxidant potential**						
TAC^14^ (µM)	101^c^	64.3^a^	66.7^ab^	75.8^b^	3.76	< 0.001
GPX^15^ (U/L)	623^a^	813^b^	782^b^	745^ab^	52.2	0.043

Dietary treatments: Control—control diet; CH—5% *Chlorella vulgaris* diet; CH+R—5% *Chlorella vulgaris* diet supplemented with 0.005% Rovabio Excel AP; CH+M—5% *Chlorella vulgaris* diet supplemented with 0.01% enzymatic mixture.

^1^Total lipids = [total cholesterol] × 1.12 + [TAG] × 1.33 + 148.

^2^TAG—triacylglycerols.

^3^HDL—high-density lipoproteins.

^4^LDL—low-density lipoproteins.

^5^VLDL—very low-density lipoproteins = 1/5 [TAG].

^6^HOMA-IR—insulin resistance index = [fasting plasma glucose] × [fasting plasma insulin] / 22.5.

^7^ALT—alanine aminotransferase (EC 2.6.1.2).

^8^AST—aspartate aminotransferase (E.C. 2.6.1.1).

^9^ALP—alkaline phosphatase (EC 3.1.3.1).

^10^GGT—gamma-glutamyltransferase (EC 2.3.2.13).

^11^IgA—immunoglobulin A.

^12^IgG—immunoglobulin G.

^13^IgM—immunoglobulin M.

^14^TAC—total antioxidant capacity.

^15^GPX—glutathione peroxidase activity. One unit of GPX is the amount of GPX that produces 1 μmol of GS-SG per min at pH = 7.6 and room temperature.

^a,b,c^Values within a row with different superscripts differ significantly at *p* < 0.05.

For immunoglobulins, IgA was kept unchanged across diets. However, IgG was increased by CH feeding, when combined with the enzymatic mixture, in comparison to the control animals (*p* < 0.001). IgM reached the lowest values in animals fed the CH diet or combined with the enzymatic mixture (*p* < 0.001) when compared to the control and Rovabio combined with CH dietary groups.

For the evaluation of serum redox status, while total antioxidant capacity (TAC) was decreased by CH feeding, with or without feed enzymes (*p* < 0.001) relative to the control, the opposite was observed for glutathione peroxidase (GPX) activity (*p* = 0.043).

### Influence of experimental diets on hepatic lipids and fatty acid composition of piglets

Table 2 shows total lipids, total cholesterol and the detailed fatty acid composition in the liver of piglets fed on CH with or without feed enzymes. Total lipids (*p* = 0.014) decreased in piglets fed CH and the enzymatic mixture, whereas cholesterol was increased in the CH group (*p* = 0.003). The sum of SFA was increased in CH, regardless of feed enzymes (*p* < 0.001), mostly due to variations in predominant fatty acids, such as C14:0 (*p* < 0.001), C16:0 (*p* < 0.001) and C18:0 (*p* < 0.001), but not C15:0 (*p* < 0.001), C17:0 (*p* < 0.001) and C20:0 (*p* < 0.001). MUFA were reduced by feeding CH in conjugation with both Rovabio and the enzymatic mixture by comparison to the control animals (*p* = 0.009). This finding was supported by the variations in C16:1*c*7 (*p* < 0.001), C18:1*c*11 (*p* < 0.001) and C20:1*c*11 (*p* < 0.001), but not by C16:1*c*9 (*p* = 0.016). Interestingly, the prevalent C18:1*c*9 was not affected by diets (*p* > 0.05). The total PUFA (*p* < 0.001), including *n*−6 PUFA (*p* < 0.001), were reduced by CH feeding with or without feed enzymes, mostly due to C18:2*n*−6 (*p* < 0.001), C18:3*n*−6 (*p* < 0.001) and C20:2*n*−6 (*p* < 0.001) results. The opposite was observed for *n*−3 PUFA (*p* < 0.001), thus contributing to a decrease in *n*−6/*n*−3 (*p* < 0.001), and PUFA/SFA (*p* < 0.001) ratios in piglets fed CH with or without feed enzymes. C20:4*n*−6 followed a decreasing trend across dietary groups with the lowest percentage in piglets fed CH with the enzymatic mixture (*p* < 0*.*001). Moreover, C18:3*n*−3 did not vary across diets (*p* > 0.05), but C22:5*n*−3 (*p* = 0.010) and C22:6*n*−3 (*p* < 0.001) increased in the CH groups.

**Table 2:** Influence of experimental diets on total lipids, cholesterol and fatty acid composition in the liver of piglets.

Item	Diets				SEM	*p* value
	Control	CH	CH+R	CH+M		
Total lipids (g/100 g)	2.12^b^	1.88^b^	1.87^b^	1.84^a^	0.065	0.014
Total cholesterol (g/100 g)	0.149^a^	0.176^b^	0.177^b^	0.178^b^	0.006	0.003
**Fatty acid composition (g/100 g FA)**						
C14:0	0.191^a^	0.254^b^	0.248^b^	0.301^c^	0.012	< 0.001
C15:0	0.841^b^	0.341^a^	0.336^a^	0.332^a^	0.058	< 0.001
C16:0	15.4^a^	21.3^b^	21.9^b^	20.6^b^	0.614	< 0.001
C16:1*c*7	0.547^b^	0.389^a^	0.388^a^	0.443^a^	0.028	< 0.001
C16:1*c*9	0.570^a^	0.805^b^	0.831^b^	0.707^ab^	0.062	0.016
C17:0	6.33^b^	2.61^a^	2.75^a^	2.66^a^	0.314	< 0.001
C17:1*c*9	0.814^b^	0.458^a^	0.470^a^	0.422^a^	0.060	< 0.001
C18:0	33.0^a^	37.7^b^	38.6^b^	40.0^b^	1.125	< 0.001
C18:1*c*9	15.3	15.0	14.6	14.4	0.412	0.463
C18:1*c*11	2.72^b^	2.03^a^	2.05^a^	2.05^a^	0.058	< 0.001
C18:2*n*−6	12.4^b^	8.53^a^	8.10^a^	8.31^a^	0.370	< 0.001
C18:2*t*9*t*12	0.123^b^	0.067^a^	0.071^a^	0.060^a^	0.008	< 0.001
C18:3*n*−6	0.111^b^	0.070^a^	0.067^a^	0.071^a^	0.007	< 0.001
C18:3*n*−3	0.058	0.066	0.067	0.064	0.004	0.263
C20:0	0.172^b^	0.097^a^	0.103^a^	0.103^a^	0.009	< 0.001
C20:1*c*11	0.364^b^	0.155^a^	0.146^a^	0.182^a^	0.017	< 0.001
C20:2*n*−6	0.758^b^	0.261^a^	0.231^a^	0.271^a^	0.036	< 0.001
C20:3*n*−6	0.150^a^	0.212^b^	0.203^b^	0.191^b^	0.009	< 0.001
C20:4*n*−6	5.83^b^	5.14^a^	4.63^ac^	4.40^c^	0.181	< 0.001
C20:5*n*−3	0.364^b^	0.155^a^	0.148^a^	0.182^a^	0.017	< 0.001
C22:0	0.128	0.167	0.162	0.193	0.028	0.413
C22:1*n*−9	0.145	0.134	0.131	0.144	0.008	0.436
C22:5*n*−3	0.223^a^	0.278^ab^	0.275^ab^	0.299^b^	0.016	0.010
C22:6*n*−3	0.519^a^	0.710^b^	0.655^b^	0.649^b^	0.023	< 0.001
C23:0	0.160^a^	0.181^ab^	0.168^a^	0.197^b^	0.007	< 0.001
Other	2.97	2.81	2.50	2.67	0.238	0.528
**Partial sums of fatty acids (g/100 g FA)**						
SFA^1^	56.3^a^	62.6^b^	64.3^b^	64.4^b^	0.793	< 0.001
MUFA^2^	20.4^b^	18.9^ab^	18.6^a^	18.4^a^	0.453	0.009
PUFA^3^	20.3^b^	15.6^a^	14.6^a^	14.6^a^	0.533	< 0.001
*n*−3 PUFA^4^	0.979^a^	1.32^b^	1.28^b^	1.28^b^	0.030	< 0.001
*n*−6 PUFA^5^	19.2^b^	14.2^a^	13.2^a^	13.2^a^	0.527	< 0.001
**Fatty acid ratios**						
*n*−6/*n*−3	19.7^b^	10.8^a^	10.4^a^	10.4^a^	0.623	< 0.001
PUFA:SFA	0.365^b^	0.250^a^	0.227^a^	0.227^a^	0.013	< 0.001

Dietary treatments: Control—control diet; CH—5% *Chlorella vulgaris* diet; CH+R—5% *Chlorella vulgaris* diet supplemented with 0.005% Rovabio Excel AP; CH+M—5% *Chlorella vulgaris* diet supplemented with 0.01% enzymatic mixture.

FA—fatty acids; SFA—saturated fatty acids; MUFA—monounsaturated fatty acids; PUFA—polyunsaturated fatty acids.

^1^Sum (C12:0, C14:0, C15:0, C16:0, C17:0, C18:0, C20:0, C22:0 and C23:0).

^2^Sum (C14:1*c*9, C16:1*c*7, C16:1*c*9, C17:1*c*9, C18:1*c*9, C18:1*c*11, C20:1*c*11 and C22:1*n*−9).

^3^Sum (C18:2*n*−6, C18:3*n*−6, C18:2*t*9*t*12, C18:3*n*−3, C18:4*n*−3, C20:2*n*−6, C20:3*n*−6, C20:4*n*−6, C20:5*n*−3, C22:5*n*−3 and C22:6*n*−3).

^4^Sum (C18:3*n*−3, C20:5*n*−3, C22:5*n*−3 and C22:6*n*−3).

^5^Sum (C18:2*n*−6, C18:3*n*−6, C20:2*n*−6, C20:3*n*−6 and C20:4*n*−6).

^a,b^Values within a row with different superscripts differ significantly at *p* < 0.05.

### Influence of experimental diets on hepatic diterpenes and pigment contents

Table 3 shows data on diterpene profile and pigments in the liver from piglets fed on CH with or without feed enzymes. Concerning the vitamin E compounds, both α- and γ-tocopherol remained unchanged by diets (*p* > 0.05). Even if chlorophyll *b* did not vary (*p* > 0.05) across dietary treatments, chlorophyll *a* reached the highest levels in piglets fed on CH with feed enzymes by comparison to the control animals (*p* < 0.001). Total chlorophylls were higher in piglets fed the combination of CH with Rovabio when compared to control animals (*p* = 0.009). Total carotenoids were increased by CH, regardless the addition of feed enzymes (*p* < 0.001). The sum of total chlorophylls and total carotenoids followed a similar pattern (*p* < 0.001).

**Table 3:** Influence of experimental diets on diterpene profile and pigments in the liver of piglets.

Item	Diets				SEM	*p* value
	Control	CH	CH+R	CH+M		
**Diterpene profile (µg/100 g)**						
α-Tocopherol	266	257	256	246	10.1	0.543
γ-Tocopherol	5.12	5.57	6.39	5.93	0.004	0.101
**Pigments (µg/100 g)**						
Chlorophyll *a*^1^	43.9^a^	76.9^ab^	104^b^	83.5^b^	9.00	< 0.001
Chlorophyll *b*^2^	87.7	123	151	125	16.6	0.066
Total chlorophylls^3^	132^a^	199^ab^	255^b^	208^ab^	23.9	0.009
Total carotenoids^4^	128^a^	218^b^	219^b^	239^b^	12.5	< 0.001
Total chlorophylls and total carotenoids^5^	260^a^	417^b^	475^b^	447^b^	30.9	< 0.001

Dietary treatments: Control—control diet; CH—5% *Chlorella vulgaris* diet; CH+R—5% *Chlorella vulgaris* diet supplemented with 0.005% Rovabio Excel AP; CH+M—5% *Chlorella vulgaris* diet supplemented with 0.01% enzymatic mixture.

^1^C*a* = 11.24 A662 − 2.04 A645.

^2^C*b* = 20.13 A645 − 4.19 A662.

^3^C*a* + *b* = 7.05 A662 + 18.09 A645.

^4^C*x* + *c* = (1000 A470 − 1.90 C*a*—63.14 C*b*)/214.

^5^(C*a* + *b*) + (C*x* + *c*).

^a, b^Values within a row with different superscripts differ significantly at *p* < 0.05.

### Principal component analyses using blood parameters and hepatic lipids and related lipid-compounds

**Figure 2:**
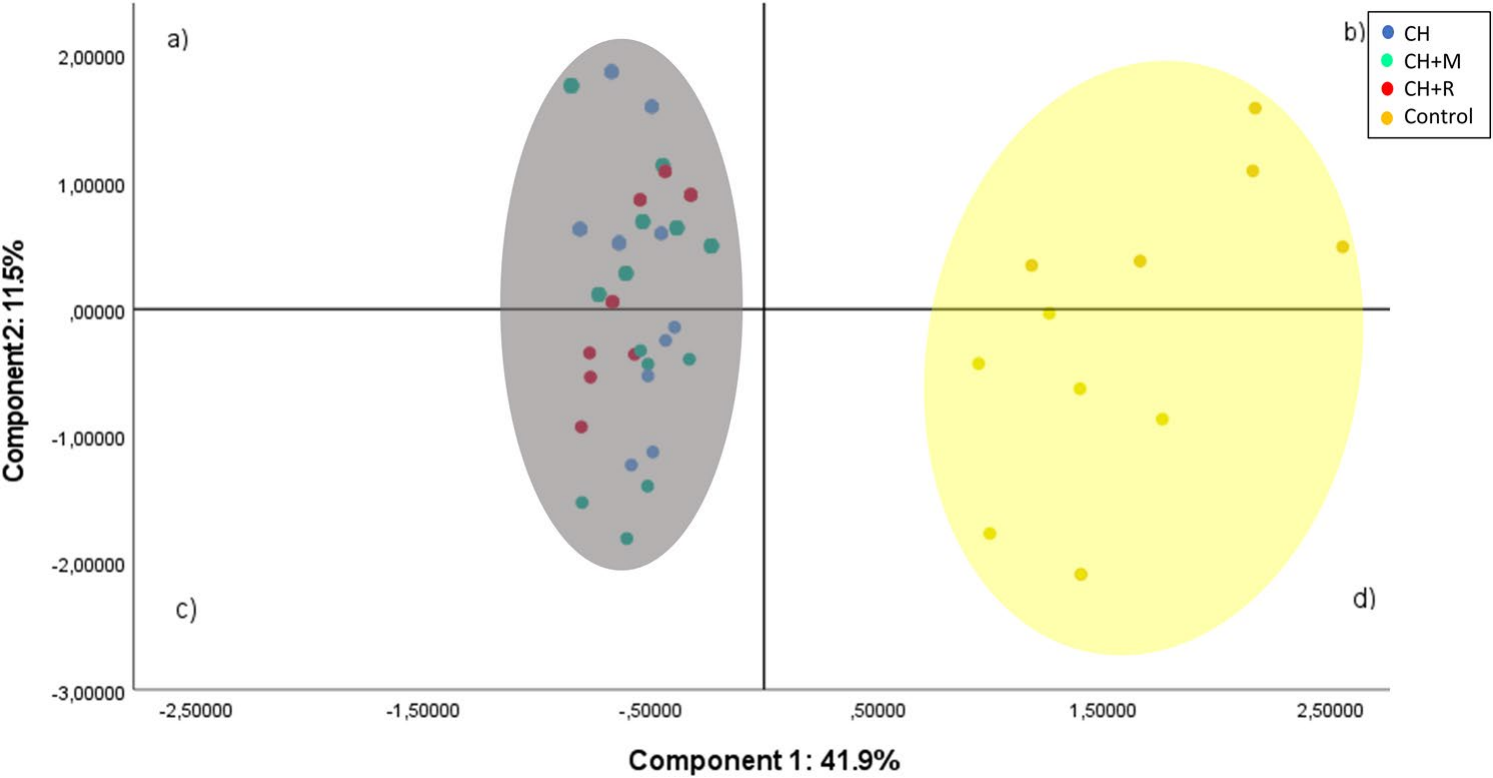
Principal component analysis (PCA) score plot using total lipids, cholesterol, fatty acid composition, diterpene profile and pigments in the liver of piglets. Dietary treatments: Control—control diet; CH—5% *Chlorella vulgaris* diet; CH+R—5% *Chlorella vulgaris* diet supplemented with 0.005% Rovabio Excel AP; CH+M—5% *Chlorella vulgaris* diet supplemented with 0.01% enzymatic mixture.

Principal component analysis (PCA) was performed with blood parameters and did not reveal a clear clustering between experimental groups (data not shown). Figure 2 illustrates the PCA output applied to a data set of 42 animal samples and 32 variables in the liver of piglets used in this trial. The first and second principal components were responsible for 53.4% of the total variance, being 41.9% for component 1 and 11.5% for component 2, respectively. As total variance explained by the first two principal components is higher than 50%, the projection of piglets’ liver samples in the plane defined by these components is shown in Fig. 2. The PCA model revealed a clear separation between the control group and the three CH-based diets (Fig. 2). The control group was confined to quadrants *b* and *d* being clearly discriminated from the other three. Microalga-based dietary groups supplemented or not with exogenous enzymes were more dispersed in quadrants *a* and *c* with no possible discrimination on the addition of feed enzymes (that is, CH, CH+R and CH+M dietary groups).

Table 4 shows the loadings for the first two principal components. Overall, component 1 was mainly characterized by positive loadings data, in particular C20:2*n*−6 (0.933), C18:2*n*−6 (0.919), C17:0 (0.889), C20:1*c*11 (0.862), C18:1*c*11 (0.856), C15:0 (0.846), C18:2*t*9*t*12 (0.834), C16:0 (− 0.810) and total carotenoids (− 0.805), while the component 2 was mainly characterized by negative loadings data, in particular C18:0 (− 0.624), C22:0 (− 0.624). C16:1*c*7 (− 0.499) and C20:3*n*−6 (0.486).

**Table 4:** Loadings for the first two principal components.

Variables	Component 1	Component 2
Total lipids	0.486	− 0.341
Total cholesterol	− 0.558	− 0.366
C14:0	− 0.624	0.370
C15:0	0.846	0.269
C16:0	− 0.810	0.363
C16:1*c*7	0.575	− 0.499
C16:1*c*9	− 0.370	0.697
C17:0	0.889	0.156
C17:1*c*9	0.710	0.377
C18:0	− 0.689	− 0.624
C18:1*c*9	0.251	0.391
C18:1*c*11	0.856	− 0.226
C18:2*n*−6	0.919	0.133
C18:3*n*−6	0.769	0.257
C18:2*t*9*t*12	0.834	0.200
C18:3*n*−3	− 0.302	0.354
C20:0	0.787	− 0.146
C20:1*c*11	0.862	− 0.238
C20:2*n*−6	0.933	− 0.099
C20:3*n*−6	− 0.615	0.486
C20:4*n*−6	0.729	0.290
C20:5*n*−3	− 0.788	− 0.133
C22:0	− 0.297	− 0.624
C22:1*n*−9	0.145	− 0.367
C23:0	− 0.445	− 0.069
C22:5*n*−3	− 0.377	0.442
C22:6*n*−3	− 0.673	0.058
α-Tocopherol	0.109	− 0.084
γ-Tocopherol	− 0.245	0.370
Chlorophyll *a*	− 0.556	− 0.080
Chlorophyll *b*	− 0.370	− 0.076
Total carotenoids	− 0.805	− 0.184

### Influence of experimental diets on the hepatic metabolome

**Figure 3:**
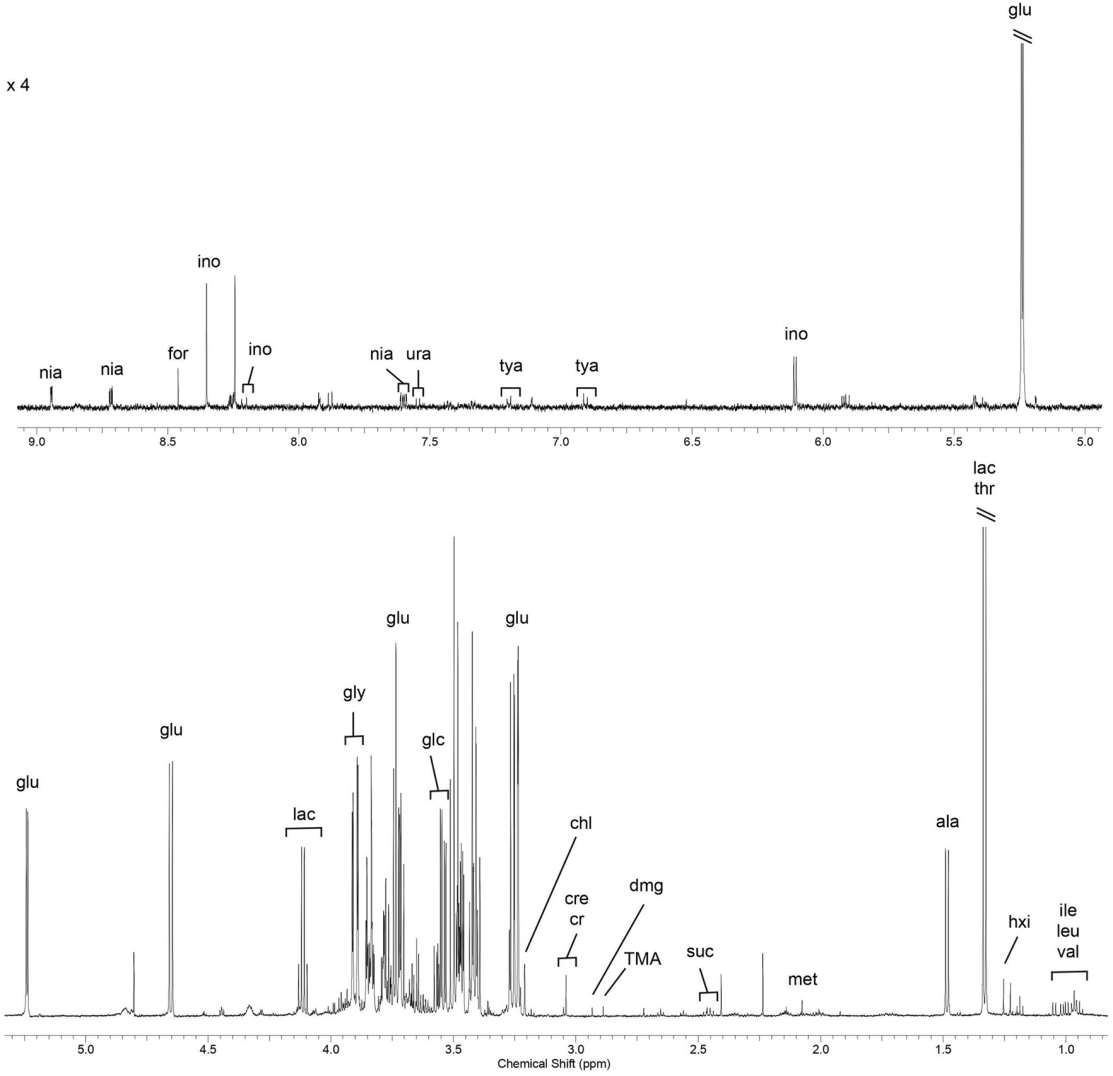
Representative NMR spectrum (^1^H 1D Presat) of liver aqueous fraction from piglets. Key: val: valine; leu: leucine; ile: isoleucine; hxi: 3-hydroxyisovalerate; lac: lactate; thr: threonine; ala: alanine; met: methionine; suc: succinate; TMA: trimethylamine; dmg: dimethylglycine; cre: creatine/creatine-P; cr: creatinine; chl: choline; glu: glucose; glc: glycerol; gly: glycine; ino: inosine; tya: tyramine; ura: uracil; nia: niacinamide/nicotinurate; for: formate.

Figure 3 shows a representative spectrum of the liver aqueous fraction from piglets with the main metabolites identified. In total, we have identified 28 metabolites that included, for instance, glucose, creatinine, and lactate. The PCA score plot computed with the bin values (Fig. S1A) showed a complete superimposition of the groups without a clear separation between them. This result is clearly indicative of a similar final metabolome profiles (general composition and concentration) between all experimental groups at the end of the experimental trial. The Partial Least Squares Analysis (PLS) model (Fig. S1B), although revealed some group separation between the experimental groups, was not validated by the quality parameters (Q^2^ < 0, 1000 permutations; *p* = 0.665). Since the permutation testing did not validate the PLS model, it is not possible to analyse the loadings plot and the variables important in projection (VIP) values of the model.

## Discussion

The use of microalgae is a relatively novel field in animal nutrition in general, and in the swine industry in particular. Thus, the literature available on this topic is scarce. Very recently, Madeira et al.^21^ studied the effects of a dietary inclusion of 10% of *Arthrospira platensis* in combination with exogenous CAZymes (commercial Rovabio and lysozyme) supplementation in weaned piglets. Despite the negative impact on piglet´s growth performance, in terms of systemic antioxidant potential and hepatic lipid metabolism, this study reported a consistent increase of total lipids, total cholesterol and LDL-cholesterol along with an improvement on antioxidant potential without variations on hepatic fatty acid content by *Arthrospira platensis*, regardless of the inclusion of exogenous enzymes^21^.

In line with these findings, we hypothesized that the dietary effect of 5% CH, alone or in combination with two exogenous enzymes (Rovabio Excel AP and a four-CAZyme mixture), might improve the immune status, antioxidant potential and change the lipid metabolism in piglets, by assessing blood biochemical parameters and hepatic fatty acids and related lipid-compounds. Dietary treatments with CH had no effect on growth performance, with no significant differences between experimental groups for live weight, ADG and FCR. Accordingly, several authors found no impact on growth parameters with 0.1–1% of CH dietary incorporation in pigs (26.6 to 53.0 kg of live weight) and weaned piglets’ diets (9.1 to 20 kg of live weight)^23,24^. Contrarily to these studies, ADFI was increased with the incorporation of CH, regardless the addition of feed enzymes, not affecting piglets’ growth performance. In the future, there is interest in confirming these results through a growth performance trial involving a large number of animals and ad libitum access to experimental diets. In our last study, we assessed the total tract apparent digestibility of nutrients and concluded that the inclusion of CH decreased the nutrients utilization by animals, with protein and fibre fractions being the most affected nutrients.

Weaning is a stressful event for piglets^25^. Changing nutrition from a milk-based diet to a cereal-based diet heavily affects the intestinal immune status and microflora^26,27^. Furthermore, changes in facilities and grouping animals of different litters can have negative consequences on physical, nutritional, immunological and behavioural status of piglets^28–30^. In terms of the immune function, while IgA did not vary among experimental groups, IgG increased in piglets fed CH-based diets and the opposite occurred for IgM, supporting their fundamental role in protecting piglets’ health. IgA, IgG and IgM are the first line of defence of the organism against infections^31^. In particular, IgG and IgM antibodies act together in immediate and long-term protection against infections, in a concerted way^32^. Upon infection, the IgM level will rise for a short time and then it will begin to drop as the IgG levels increase, protecting the organism in the long-term^32^. In fact, CH polysaccharides, carotenoids and pigments have already shown strong immunomodulatory activities and recent evidence demonstrated the prebiotic effect of CH powder in treated rats^33^. The main action of prebiotics is to stimulate growth and/or activate metabolism of protective bacteria in the intestinal tract, thus benefiting intestinal microbiome, and ultimately, piglets’ health. In line with this, lymphocytes were also increased in piglets fed CH-based diets and even more when combined with the enzymatic mixture. Taken together, these positive variations reflect a boost on the immune response stimulated by CH that likely assures piglets’ survival at the critical period of weaning.

Blood parameters have been increasingly used as body condition indicators as they provide valuable information on the physiological condition of the animal^34^. Herein, the lipid profile of piglets was largely influenced by dietary treatments. Cholesterol is partially obtained from the diet, by consumption of animal-derived products, and from de novo biosynthesis in the liver^35^. Even if a pattern of increase was promoted by CH for total cholesterol, LDL-cholesterol and VLDL-cholesterol, not in agreement with previous studies^33^, these variations were positively counterbalanced by a rise in HDL-cholesterol in piglets fed the exogenous enzymes, putatively leading to healthy cardiovascular functions^36,37^. The reverse cholesterol transport is the mechanism by which the organism removes excess cholesterol from peripheral tissues and delivers it to the liver, where it will be redistributed to other tissues or removed from the organism, being HDL-cholesterol the main lipoprotein responsible for this process. Total lipids and TAG reached also higher values in piglets fed CH diets with or without feed enzymes. Notwithstanding, it should be underlined that the values found for systemic lipemia were not very far from the ones obtained previously by our research team^21^. These variations do not seem to promote, in the long-term, fatty liver, which is a serious pathophysiological condition associated with several human metabolic disorders, in particular obesity, diabetes and hyperlipidaemia^38,39^.

Despite the variations observed for aminotransferase activities, it is worth noticing that the levels found are close to the reference values for pigs, which are 31–58 U/L for ALT, 32–84 U/L for AST and 10–52 U/L for GGT^40^. In view of these results, there is no clear evidence of CH toxicity. Urea and creatinine reached the highest levels in piglets fed the combination of CH with the enzymatic mixture, in agreement with the same range of levels variations found for creatinine by Madeira et al.^21^ with *Arthrospira platensis* and lysozyme. Glucose was unaffected by CH-based diets, but insulin decreased with the enzymatic mixture pointing towards a positive effect on glycemia homeostasis by degrading enzymes, in virtue of a tendency for glucose decrease (albeit with no statistical significance) in this same experimental group. Insulin is a well-known stimulator of lipogenesis^41^ and stimulates fatty acid synthesis in the liver with formation and storage of triacylglycerols^42^. Nevertheless, the values found for insulin resistance index were within the normal physiological range, below 2.4^43^.

The accurate assessment of redox status in vivo of the organism can only be determined by the measurement of total antioxidant capacity^44^. Although the concentration of serum antioxidant components can be measured individually, these measurements may be time- and cost-consuming as well as labour intensive^45^. In addition, it may not accurately reflect the total antioxidant status^46^. CH with or without exogenous enzymes decreased TAC in serum, which is not consistent with the increase on hepatic total carotenoids and total chlorophylls. Carotenoids and chlorophylls are natural lipophilic pigments with antioxidant behaviour and free radical-scavenging properties, especially for chlorophylls, that are present in the diet^47^. In the present study, the decrease of TAC variation in serum was accompanied by a consistent increase in GPX activity in piglets fed CH-based diets suggesting a compensatory mechanism to avoid imbalance of oxidative stress homeostasis. GPX, an important antioxidant enzyme plays a key role in protecting haemoglobin, red blood cell enzyme activity and biological cell membranes against oxidative damage^48^ with the highest activity found in the liver and red blood cells^49^.

In pigs, fatty acid composition of skeletal muscle, subcutaneous fat and liver is much more modulated by the pig genotype than by the dietary lipid level^50^. Total SFA was increased by CH, whereas MUFA was reduced by the microalga in conjugation with either Rovabio or the enzymatic mixture, but not by the microalga itself. On the positive side, *n*−6 PUFA were reduced by CH with or without feed enzymes, most at the expenses of C18:2*n*−6, C18:3*n*−6, C20:2*n*−6 and C20:4*n*−6, this last fatty acid being responsible for overproduction of prothrombotic and pro-inflammatory eicosanoids, thromboxanes and leukotrienes^51^. The inverse was observed for *n*−3 PUFA, in particular for the valuable DPA and DHA fatty acids^16^, impacting positively on *n*−6/*n*−3 ratio. *n*−3 fatty acids are substances of particular interest in animal feeding due to their anti-microbial and antioxidant action, as well as their biofortification ability of animal products^52^. Moreover, the enrichment in *n*−3 PUFA in the liver has been linked to positive events, such as downregulation of PUFA oxidation-associated genes expression, diminished lipid peroxidation and enhanced antioxidant properties^53^.

The impact of CH dietary incorporation, with or without feed enzymes, on hepatic levels of tocopherols and pigments was also determined. Vitamin E is known as the major free radical chain terminator in the lipophilic environment^54^. Among the vitamin E compounds, α-tocopherol was the major vitamin E homologue in all dietary groups, whereas γ-tocopherol was the minor, which strongly agree with Madeira et al.^21^. Contrarily to what was demonstrated for *Arthrospira platensis*^21^, there was no negative impact of CH and carbohydrases on vitamin E compounds. On the contrary, pigments were overall increased by CH, with or without feed enzymes. The rise of total chlorophylls and total carotenoids contents in the liver is thought to be a key indicator of their respective dietary bioavailability. Chlorophylls and carotenoids are powerful dietary antioxidants^47^, which are extremely important for human and piglets’ health^55^.

The discriminant analysis herein presented, a PCA based on the relationship among all hepatic variables, showed a clear separation of dietary treatments with or without CH. Interestingly, for the control group, and in contrast with CH-based diets, a higher dispersion pattern of animal cases was observed. It remains to be elucidated what might be the cause for such observation.

Finally, and concerning the metabolomics analysis, the PCA applied to the liver aqueous metabolites showed a complete superimposition of the four experimental groups. Moreover, the PLS model, although presented some group clustering, was not validated by the quality parameters. These results clearly indicate the existence of very similar metabolite profiles for the four experimental groups. Such metabolite profiles suggest that the inclusion of CH in the diet, supplemented or not with exogenous enzymes, had a minimal effect on the overall hepatic intermediate metabolism. These results are similar to other studies on the effects of diet in the metabolite profiles of several swine tissues^56–58^ where only minor differences were noticeable in the hepatic metabolome. This could be considered expectable given the fact that these animals, from a physiological standpoint, were in similar physiological stages, at a young age and still growing. On the contrary, when the liver metabolome of piglets with limited growth is compared to that of control growing piglets^59^, the number of affected metabolites and metabolic pathways are increased. Nevertheless, it is noteworthy to mention that the NMR-based metabolomics approach was a useful tool to, not only complement datasets obtained from the other techniques, but also to evaluate the overall dietary influence on the liver metabolome.

## Conclusion

The dietary inclusion of 5% CH supplemented or not with enzymes, the commercial Rovabio and the pre-selected four-enzyme mixture, had no impact on the growth performance of piglets, although systemic antioxidant potential and hepatic lipid metabolism were affected. The first line of antioxidant defence through GPX activity and hepatic *n*-3 PUFA contents, in particular the beneficial DHA, were increased by the microalga inclusion in the feed. In piglets fed CH-based diets, the interaction observed between IgG increase and IgM decrease, along with lymphocytes exacerbation, reflected a boost on the immune response promoted by CH that likely assures piglets’ survival at the critical post-weaning phase.

Considering the findings obtained in this study, particularly those concerning long-term immune reinforcement, our data indicate health benefits of CH used as feed ingredient in piglets’ nutrition, without negatively impacting animals’ performance. Nevertheless, further research with higher incorporation levels of CH in piglets’ diets are suggested, in order to maximize both the sustainability of swine diets and the health promoting effects of dietary CH incorporation. The dietary supplementation with exogenous carbohydrases does not seem to be necessary for feeding piglets with CH-based diets at this level of incorporation. Although, testing higher levels of microalgae incorporation can be interesting to verify the effect of this supplementation with feed enzymes, and also to ascertain the cost-effective to their use.

## Methods

### Design trial and experimental treatments

All the procedures used in this animal experiment were revised by the Ethics Commission of ISA and approved by the Animal Care Committee of the National Veterinary Authority (Process Number 0421/2017, Direção Geral de Alimentação e Veterinária, Portugal). All methods were carried out in accordance with the European Union legislation (2010/63/EU Directive) and are reported following the ARRIVE guidelines 2.0 (https://arriveguidelines.org/arrive-guidelines).

Forty-four castrated male piglets from Large White × Landrace sows crossed with Pietrain boars weaned at 28 days of age and with initial live weight of 11.2 ± 0.46 kg were selected. Details on the animal experiment were previously described by Martins et al.^60^. Briefly, animals were housed individually in metabolic cages and had ad libitum access to water and restricted access to diets (to perform a digestibility study; data not shown). Following a two-day adaptation period, two animals were excluded from the trial. The piglets were randomly distributed into one of 4 experimental groups: control (corn and soybean meal-based diet, *n* = 11), CH (control diet with 5% CH, *n* = 10), CH+R (control diet with 5% CH plus 0.005% Rovabio Excel AP, *n* = 10), and CH+M (control diet with 5% CH plus 0.01% of a four-CAZyme mixture, *n* = 11). CH microalga was purchased from Allmicroalgae—Natural Products SA (Pataias, Portugal) and incorporated as freeze-dried powder into the diets. CH chemical details were previously described by Coelho et al.^61^. The level of CH incorporation (5%) followed this previous study, maintaining the main objective of our line of investigation (which is to test microalgae as ingredient). Rovabio Excel AP was incorporated into the diets at 0.005%, as recommended by the manufacturer. The four-CAZyme mixture composed by an exo-β-glucosaminidase, an alginate lyase, a peptidoglycan N-acetylmuramic acid deacetylase and a lysozyme was pre-selected and tested in vitro for efficient degradation of CH cell walls^19^. The homogenous distribution of enzymes was guaranteed by a pre-mixture with a feedstuff excipient and the microingredients. Table 5 shows diets composition. The chemical composition of diets was described by Martins et al.^60^. To determine ADFI, ADG and FCR, feed supplied and refusals were weighed daily and piglets were weighed weekly.

**Table 5:** Ingredients and feed additives of the experimental diets (g/kg).

Ingredients	Control	CH	CH+R	CH+M
Wheat	439	440	440	440
Corn	150	150	150	150
Soybean meal 48	250	200	200	200
Whey powder	100	100	100	100
Sunflower oil	30	30	30	30
*Chlorella vulgaris*	0	50	50	50
Rovabio® Excel AP	0	0	0.05	0
Four-CAZyme mixture^1^	0	0	0	0.1
L-Lysine	5	5	5	5
DL-Methionine	1	1	1	1
L-Threonine	1	1	1	1
Calcium carbonate	5	6	6	6
Dicalcium phosphate	13	12	12	12
Sodium chloride	3	2	2	2
Vitamin-mineral complex^2^	3	3	3	3

Dietary treatments: Control—control diet; CH—5% *Chlorella vulgaris* diet; CH+R—*Chlorella vulgaris* diet supplemented with 0.005% Rovabio Excel AP; CH+M—*Chlorella vulgaris* diet supplemented with 0.01% enzymatic mixture.

^1^exo-β-glucosaminidase, an alginate lyase, a peptidoglycan N-acetylmuramic acid deacetylase and a lysozyme (CPE1314).

^2^Premix provided per kg of complete diet: vitamin A, 6500 UI; vitamin D3, 1500 UI; vitamin E, 15 mg; vitamin K3, 1 mg; vitamin B1, 1 mg; vitamin B2, 3 mg; vitamin B6, 2 mg; vitamin B12, 0.02 mg; pantothenic acid, 10 mg; nicotinic acid, 15 mg; folic acid, 0.5 mg, biotin, 0.03 mg; betaine, 115 mg; vitamin C, 20 mg; Copper, 100 mg; iron, 100 mg; iodine, 0.5 mg; manganese 50 mg; selenium, 0.15 mg; zinc, 100 mg; butylated hydroxytoluene, 3 mg.

### Slaughter and sampling

After an experimental period of 15 days, with a live weight of 23.1 ± 2.56 kg, all animals were slaughtered, following the standard procedures of commercial abattoirs, using electrical stunning, followed by exsanguination. Blood samples were collected with anticoagulant EDTA and analysed for haematology on the same day; for all the other parameters, blood samples were centrifuged at 1500 g for 15 min to obtain serum, and stored at − 20 °C, until analysis. Liver samples were collected, vacuum packed and stored at − 20 °C for fatty acid composition and pigment analysis. Samples used for metabolomics were snap-frozen in liquid nitrogen and stored at − 80 °C until further analysis.

### Determination of blood parameters

As previously described by Madeira et al.^21^, red blood cells, white blood cells and thrombocytes counts were performed using Sysmex XN-10 (Sysmex Corporation, Kobe, Japan) analysers. The red blood cells count was measured using the impedance variation method after hydrodynamic focusing. For white blood cells differential counting (%), the blood smears were discoloured with the May-Grünwald-Giemsa technique. The haemoglobin concentration was measured by photometry, at 522 nm, with sodium lauryl sulphate as reagent.

The determination of total cholesterol, HDL-cholesterol, LDL-cholesterol, TAG, phospholipids, total protein, urea, creatinine and glucose concentrations, aspartate aminotransferase (AST), alanine aminotransferase (ALT), alkaline phosphatase (ALP) and gamma-glutamyltransferase (GGT) was performed in a Modular Hitachi Analytical System (Roche Diagnostics, Mannheim, Germany), through commercial kits (Roche Diagnostics, Basel, Switzerland). For VLDL-cholesterol and total lipids, Friedewald et al.^62^ and Covaci et al.^63^ formulas were applied, respectively. The concentration of insulin was determined in serum using the Porcine Insulin RIA kit (PI-12 K; Linco Research, Millipore, Billerica, MA, USA). The degree of insulin resistance was calculated by the homeostasis model assessment using the formula described by Matthews et al.^64^: insulin resistance index (HOMA-IR) is equally to fasting serum glucose (mmol/L) multiplied by fasting serum insulin (mU/L) and divided by 22.5. The immunoglobulin profile (IgA, IgG and IgM) was defined by immunoturbidimetry.

The total antioxidant capacity (TAC) was measured in serum through the QuantiChrom Antioxidant Assay Kit (https://bioassaysys.com/datsheet/DTAC.pdf, Bioassay Systems, Hayward, CA, USA). The glutathione peroxidase (GPX) activity was assessed in serum by the EnzyChrom Glutathione Peroxidase Assay Kit (https://www.bioassaysys.com/datasheet/EGPX.pdf, Bioassay Systems). One unit of GPX is the amount of GPX that produces 1 μmol of glutathione disulphide (GS-SG) per min at pH = 7.6 and room temperature.

### Hepatic total fat content and fatty acid profile

After liver samples freeze drying (at − 60 °C and 2.0 hPa, Edwards Modulyo freeze drier, Crawley, UK), total lipids were gravimetrically quantified in duplicate, following Folch et al.^65^ method, using dichloromethane and methanol, as reported by Carlson^66^. Subsequently, the fat residue was resuspended in dry toluene and subjected to successive alkaline and acid transesterification reactions to convert fatty acids into fatty acid methyl esters (FAME)^67^. FAME separation was performed by gas–liquid chromatography with flame ionization detector (GC-FID HP7890A Hewlett-Packard, Avondale, PA, USA), as previously described by Madeira et al.^21^. Fatty acids were expressed as percentage of total fatty acids, after identification by their retention times and quantification, using heneicosanoic acid (C21:0) as internal standard and by converting the relative peak areas into weight percentages.

### Hepatic cholesterol, diterpene profile and pigments determination

Total cholesterol and diterpene profile were determined in duplicate in liver samples, as previously described by Prates et al.^68^. After a direct saponification of samples, one aliquot of the *n*-hexane layer was filtered before run into an HPLC system (Agilent 1100 Series, Agilent Technologies Inc., Palo Alto, CA, USA). Total cholesterol and β-carotene were detected using UV/Vis photodiode array detector (λ = 202 nm and λ = 450 nm, respectively), and tocopherols and tocotrienols using fluorescence detector (excitation at λ = 295 nm and emission at λ = 325 nm). The concentration of total cholesterol, β-carotene and vitamin E homologues in hepatic samples was quantified using a standard curve of peak area *vs*. concentration.

The quantification of pigments in hepatic samples was performed using Teimouri et al.^69^ protocol with slight adjustments. Briefly, hepatic samples were incubated at room temperature with acetone overnight, under agitation, and in the dark. After extraction, samples were centrifuged at 1500 g for 5 min and analysed by UV/Vis spectrophotometry (Ultrospec 3100; Amersham Biosciences, Little Chalfont, UK), at 662 nm for chlorophyll a, at 645 nm for chlorophyll b, and at 470 nm for total carotenoids. The pigment contents were calculated using Hynstova et al.^70^ equations.

### Hepatic NMR-metabolomics analysis

Liver tissue was powdered without thawing in liquid nitrogen. The extraction of the aqueous metabolites from the liver ground powder was performed following the chloroform/methanol method, as previously described by Palma et al.^71^ Then, the aqueous fraction samples were resuspended in phosphate buffer (1.75 M K_2_HPO_4_ (anhydrous); 1.24 mM sodium formate; 5.0 M 3-(trimethylsilyl) propionic-2,2,3,3-d4 acid sodium salt (TSP); pD 7.40; in ^2^H_2_O) and 99.8% ^2^H_2_O. Proton-decoupled ^1^H NMR spectra were obtained using a Varian VNMRS 600 MHz (Agilent, Santa Clara, CA, USA) spectrometer equipped with a 3 mm ^1^H(X)-PFG inverse configuration probe. A ^1^H-Presat pulse sequence was acquired for each sample (spectral width 7 kHz; acquisition time 4 s; saturation delay 3 s; relaxation delay 4 s; 6 scans; at 298 K). All spectra were processed in the ACD/NMR Processor Academic Edition from ACD\Labs 12.0 software (Advanced Chemistry Development, Inc.) applying: zero-filling to 65 k, line broadening of 0.2 Hz, phasing, baseline correction and the chemical shifts were referenced to the TSP peak at 0 ppm (or any other internal standard). Spectral binning was performed in ACD/NMR Processor Academic Edition using uniform binning with a 0.04 ppm width from − 0.5 to 10 ppm. Regions for water (4.70–5.15 ppm) and TSP (− 0.5 to 0.25 ppm) were excluded.

The multivariate analysis was performed with the bin values, using MetaboAnalyst 4.0 software (https://www.metaboanalyst.ca) for Principal Component Analysis (PCA) and Partial Least Squares analysis (PLS). For the PLS analysis, Q^2^ (predictive ability of the model), R2 (goodness of the fit), and the *p* value of the permutation test (1000 permutations) were considered as the quality parameters for each model. PLS models were accepted as valid for Q2 above 0.5 and *p* value < 0.05^72^. For both PCA and PLS models, the ellipses in the score plots were drawn using a 95% confidence level.

### Statistical analysis

Using SAS software package (version 9.4, SAS Institute Inc., Cary, NC, USA), all data were analysed by one-way analysis of variance (ANOVA) selecting the General Linear Model (GLM) procedure. Normal distribution and variance homogeneity were verified for all data through the Shapiro–Wilk and Levene tests, respectively. The statistical model considered the piglet as the experimental unit and the dietary treatment as the single effect. To determine the significant effects of dietary treatments, least-squares means for multiple comparisons were generated by the PDIFF option and adjusted with the Tukey–Kramer method. The results were considered significantly different when *p* ≤ 0.05. The PCAs were performed with blood parameters and all hepatic variables using the SPSS Statistics for Windows (IBM Corp. released 2020, version 27.0, Armonk, NY, USA).

## Supplementary Information

Supplementary Information 1.

## Data Availability

All data generated in this study are included in the published article. The datasets generated during the current study are available from the corresponding author on demand upon reasonable request. The raw NMR spectra obtained during the current study have been uploaded to the Zenodo repository (https://zenodo.org) with the reference 5,822,944, and 10.5281/zenodo.5822944.
