# Supplementary figures and images for: Impact of dietary Chlorella vulgaris and feed enzymes on health status, immune response and liver metabolites in weaned piglets
Source: Sci Rep. 2022 Oct 7;12:16816. doi: 10.1038/s41598-022-21238-9 (PMC9546893; doi:10.1038/s41598-022-21238-9)

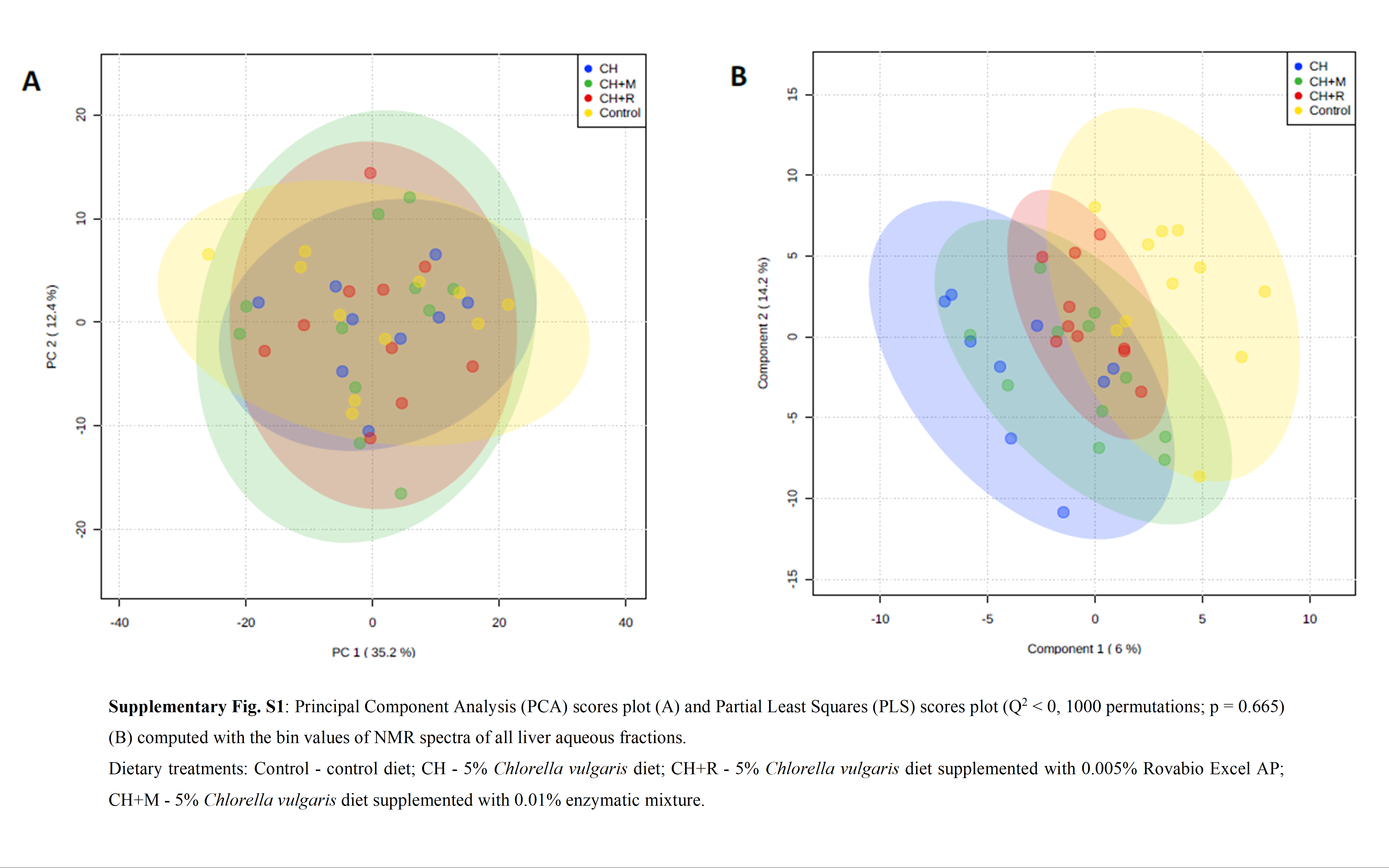

Supplement: Supplementary file 1 — Supplementary Information 1. [file 41598_2022_21238_MOESM1_ESM.tif]
